# Beliefs about Medicines and the Level of Intentional Non-Adherence to Treatment among Patients with Multiple Sclerosis Treated with First-Line Drugs

**DOI:** 10.3390/jcm13010182

**Published:** 2023-12-28

**Authors:** Aleksandra Kołtuniuk, Justyna Chojdak-Łukasiewicz

**Affiliations:** 1 Department of Nursing and Obstetrics, Wroclaw Medical University, 50-367 Wroclaw, Poland; 2 Department of Neurology, Wroclaw Medical University, 50-367 Wroclaw, Poland; justyna.chojdak-lukasiewicz@umw.edu.pl

**Keywords:** multiple sclerosis, treatment adherence, disease-modifying therapy, beliefs

## Abstract

Introduction: Multiple sclerosis (MS) is a chronic inflammatory, demyelinating and neurodegenerative disease of the central nervous system. MS has no curable disease but drug modifying therapy (DMT) can delay the long-term disability progression of the disease. The effectiveness of MS treatment depends on the patient’s adherence to therapy. Aim: This study evaluated the level of intentional non-adherence and the relationship between beliefs about medication and the level of intentional non-adherence to treatment of patients with multiple sclerosis. Material and methods: A group of 146 patients with relapsing–remitting MS were included. To assess different aspect of adherence, the Intentional Non-Adherence Scale (INAS) was used. For evaluating patients’ beliefs and opinions regarding medication, the Beliefs about Medicines Questionnaire (BMQ) was used. Results: The mean total INAS score was 51.41 ± 27.83 points. Patients were most concerned about the necessity to take medication and least concerned about the harm caused by medication. The overuse and harm domains of the BMQ were significantly correlated with INAS scores (*p* < 0.05). Conclusions: Independent determinant of intentional non-adherence was overuse.

## 1. Introduction

Adherence with prescribed medication is an essential factor determining successful treatment. Based on its definition, adherence is the degree to which a patient’s behaviour is consistent with the recommendations provided in their treatment plan [1]. Adherence is presented as the number of drug doses taken compared to the prescribed dose and can be measured directly or indirectly (e.g., by a self-report scale or an interview) [2].

Multiple studies have identified factors affecting adherence. According to the WHO, the factors have been classified into five groups: social and economic factors; healthcare system factors; medical condition-related factors; treatment-related factors; and patient-related factors [3].

Non-adherence to drugs is a complex problem that affects many therapeutic areas. It is a particular problem in patients with diagnosed chronic conditions. Approximately 30 to 50% of patients do not follow their prescribed medications consistently [4]. Non-adherence to treatment can be classified into two categories, intentional or unintentional, according to the patient’s perspective.

Unintentional adherence is a passive process that is characterised by not taking medication (due to forgetfulness or carelessness). It may result from cognitive impairment or circumstances not directly controlled by the patient. Patients who have been taking medication for a long time often unintentionally stop taking their medication regularly due to routine or habit [5,6].

Intentional non-adherence involves a deliberate decision not to take prescribed medication as directed. Patients, often after weighing the costs and benefits of treatment, make the decision to stop taking their medicines or stop adhering to treatment recommendations. Factors influencing intentional non-adherence include including polypharmacy, negative perceptions about treatment, concerns about ineffectiveness or adverse effects of medication, lack of belief in the possibility of recovery, feeling healthy and symptom-free, and others’ opinions about a specific medication [7,8]. To assess intentional vs. non-adherence, validated questionnaires are used. Weinman et al. developed the Intentional Non-Adherence Scale (INAS) tool, which is used to assess intentional non-adherence to prescribed therapy [9]. The Beliefs About Medication (BMQ scores) provide information about patients’ actual medication taking behaviour [8].

The aim of study was to evaluated the level of intentional non-adherence among patients with multiple sclerosis and indicate the relationship between beliefs about medication and the level of intentional non-adherence to treatment in patients with MS.

Current management strategies are focused on treatment acute attacks, symptomatic therapy and disease-modifying therapy (DMT). DMTs modify the course of the disease and protect against progression by affecting the immune system [10]. Adherence to treatment in multiple sclerosis is critical for successful therapy. Non-adherence in MS has impacts on the progression of disease and the quality of life and is also associated with poorer outcomes and higher medical healthcare use.

## 2. Material and Methods

### 2.1. Study Design

The investigators used a cross-sectional study design with a questionnaire-based survey. The research was carried out in the Department of Neurology, Wroclaw Medical University, from September to October 2022. Participants in this study were recruited from a group of patients under the constant outpatient care. A total of 148 patients with relapsing–remitting MS were enrolled.

All patients met the following inclusion criteria:

(1) Fulfilled the McDonald’s criteria;
(2) Treatment with DMT therapy;
(3) Signed informed consent.

Patients responded to traditional self-administered pencil-and-paper questionnaires, which were designed to be completed in approximately 10 min during each check-up visit. Data collection also included demographic data. Medical data (clinical characteristics, EDSS score and type of treatment) were collected from the hospital database.

### 2.2. Questionnaires

In the study, self-reported questionnaires investigating the factors related to treatment adherence were used.

The evaluation comprised the following measures:

1. The Intentional Non-Adherence Scale (INAS) is a scale to assess intentional non-adherence to prescribed medications [9]. The questionnaire consists of 22-item scale scored on a 5-point Likert scale (1 = strongly disagree, 2 = disagree, 3 = neutral, 4 = agree, 5 = strongly agree). The final score ranges between 22 and 110, and higher scores indicate poorer adherence.
2. Beliefs about Medicines Questionnaires (BMQ) [11,12].

Beliefs about medication were measured using the BMQ, which assesses patients’ medication beliefs in general and also assesses their personal views about the necessity of prescribed medication for controlling their illness and their concerns about the potential adverse consequences of taking their prescribed medication. It is a validated questionnaire consisting of 18 questions divided into 2 parts (BMQ General and BMQ Specific). Participants indicate their degree of agreement with each statement on a 5-point Likert scale, which ranged from 1 = strongly disagree to 5 = strongly agree. The scores obtained for individual items are totalled to give an overuse, harm, necessity and concerns scale score. Total scores for the overuse and harm scales range from 4 to 20, while total scores for the necessity and concerns scales range from 5 to 25. Higher scores in each subscale represent a negative perception of medication. The validity of the BMQ in assessing medication adherence has been demonstrated in various diseases and populations [11,13].

### 2.3. Ethical Consideration

The research was approved by the Bioethics Committee of Wroclaw Medical University (approval no. KB 175/2022). Patients signed informed consent forms for data collection. Participation was voluntary and anonymous, and all patients were informed about the purpose, methods, course of the study and about their right to decline or discontinue their participation. The study was conducted according to the Helsinki Declaration.

### 2.4. Statistical Methods

The analyses were performed using R software (Vienna, Austria), version 4.0.1. For the measurable variables, the arithmetic mean (M), median (Me), standard deviation (SD), extreme values (Min and Max) and quartile were calculated; for the non-measurable variables, the percentages (%) were calculated. Comparisons of qualitative variables in groups were conducted with the chi-square test (with Yates’ correction for 2 × 2 tables) or with Fisher’s exact test (when low expected values occurred). Comparisons of quantitative variables in two groups were conducted with the Mann–Whitney test. Correlations between quantitative variables were assessed with Spearman’s correlation coefficient. Multivariate analysis of the simultaneous impact of many independent variables on one quantitative dependent variable was made by means of linear regression. The 95% confidence intervals were reported along with the regression parameters. Analyses were conducted at a 0.05 level of significance.

## 3. Results

### 3.1. Sociodemographic and Clinical Characteristics

The study included 148 patients with diagnosed MS (100 females, 48 males, aged 37–50, mean 43.9). The mean EDSS score was 2.38 ± 1.27. The mean duration of the disease was 12.07 ± 6.35. All patients were treated with DMTs: 61 (41.22%) with the self-injectable form (subcutaneous or intramuscular) and 87 (58.78%) with oral drugs. The mean treatment duration was 6.22 ± 3.67. The most debilitating symptoms of MS reported by the patients were fatigue (70.95%), balance problems (56.76%) and vision disturbances (49.32%). The demographics and clinical characteristics of the study are summarised in Table 1.

### 3.2. Level of Intentional Non-Adherence (INAS Scores) and Beliefs about Medication (BMQ Scores)

The mean total INAS score was 51.41 ± 27.83 points; 82 (55.41%) patients had a low level of intentional non-adherence and 66 (44.59%) patients had a high level of intentional non-adherence. The results of the BMQ subscales are presented in Table 2. The patients obtained 18.30 ± 3.41 scores on necessity, 14.17 ± 3.29 scores on concerns, 10.69 ± 2.55 scores on harm and 11.28 ± 2.5 scores on overuse. Respondents were most concerned about the necessity to take medication (18.30) and least concerned about the harm caused by medication (10.69).

### 3.3. Correlation between Non-Adherence and Beliefs about Medication

**Figure 1:**
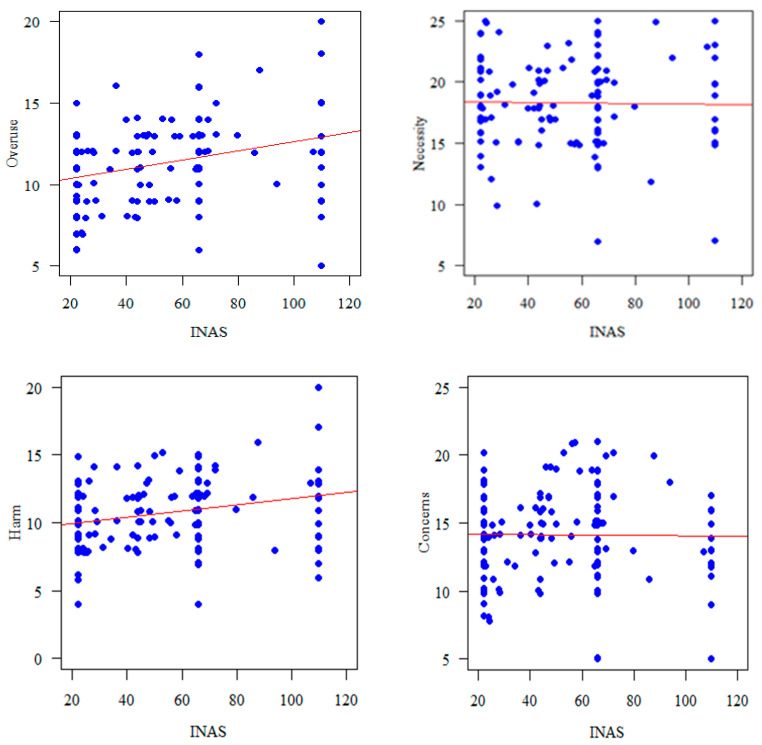
Correlation of BMQ subscales with INAS.

The overuse (r = 0.330, *p* < 0.0010) and harm (r = 0.27, *p* = 0.001) subscale of the BMQ were significantly correlated with INAS scores (Figure 1).

The impact of different variables in the presence of non-adherence behaviour was studied in two logistic regression models. The variables for correlation with intentional non-adherence are shown in Table 3 and Table 4, with linear regression indicating no relationship between the variables. The multivariate linear regression model showed that beliefs that medicines are overused by doctors is an independent predictor of higher intentional non-adherence levels (β = 4.204, *p* = 0.011).

## 4. Discussion

Adherence to therapeutic recommendations in patients with multiple sclerosis is the primary determinant for effective therapy with DMTs. The studies have shown that adherent patients with multiple sclerosis (MS) have fewer relapses, visit healthcare providers less often and have reduced medical costs compared to non-adherent patients [14,15]. Despite these benefits of long-term outcomes, research has shown that non-adherence is a common problem in patients with MS [16]. The level of adherence in MS varies widely between 41% and 93% and depends on the assessment method and medication type [17,18,19]. In a study, Hansen et al. [20] reported that the adherence rates ranged between 30% to 40% two years after starting use of MS-modifying treatment. Burks et al. [21] carried out an analysis on a real-world cohort of more than 12,000 patients with MS showing that nearly 40% of patients were not adherent to DMT.

The unintentional non-adherence (forgetting to take medication) is associated with demographics, age and comorbidities [22]. A previous study [18] focused mainly on unintentional causes of non-adherence in patients with MS. It has been shown that, due to forgetting or misunderstanding instructions, a large group of patients at some point make unintentional errors in their medication regimen. Cognitive impairment is one of the causes of why patients did not comply with the treatment. Problems with episodic memory, concentration and executive functions are the main reason for not taking the drug due to forgetfulness [23]. Also, depression in MS is associated with non-adherence [24,25].

Motivational factors for adherence behaviour are diverse, included drug-related aspects and psychological factors such as illness beliefs or factors concerning treatment decision making [26].

Most views people possess about medications commonly fall into four different categories concerning the necessity of the given medication, the possible adverse effects and reactions that come from its use, a general idea of the overuse or over-prescription of a medication and an idea of medications causing harm in general [11].

In this study, patients were most concerned about the necessity of taking their medication (18.30) and least concerned about the harm caused by the medication (10.69). In a study conducted by Thach et al. [27], patients reported perceived necessity on the moderate level (mean ± SD, 18.3 ± 3.8) and perceived concern on the low level (mean ± SD, 10.6 ± 3.7). Pust et al. [28] has shown that that patients treated in the second-line obtained higher results on the BMQ Specific Necessity Beliefs domain and also on the BMQ Specific Concerns Beliefs domain compared to patients treated in the first-line.

According to the Necessity–Concerns Framework, beliefs about the necessity of taking medications and concerns about the potential side effects of medications are key beliefs that influence medication adherence [29]. Research to date has shown that beliefs about medicines have been shown to be an important factor influencing adherence [30,31,32,33]. Pust et al. [28] has shown that first-line patients, with less necessity beliefs for treatment, are at risk of being non-adherent patients. However, studies conducted by Thach et al. [27] and Strosova et al. [34] did not confirm these correlations.

In this study, we describe the relationship occurring between beliefs about medications and the levels of intentional non-adherence to treatment in patients with multiple sclerosis.

Generally, intentional non-adherence, including missing or altering doses, stems from rational patients’ decisions which depend on their beliefs about treatment [35].

In a study by Bischoff et al. [36], the main reasons for non-adherence in group patients treated with first-line drugs were ‘‘subjective’’ reasons, such as fear of injection, doubt about efficacy, loss of efficacy and size effect and non-adherence in MS patients receiving second-line drugs was a perceived lack of effectiveness.

In our study, the mean rate of intentional non-adherence was 51.41 ± 27.83 (55.41% of patients had a low level of intentional non-adherence and 44.59% of patients had a high level of intentional non-adherence). In a study by Świątoniowska et al. [30], the mean (SD) total INAS score was 47.28 (19.12) points, and 72.33% of patients had a low level of intentional non-adherence and 27.67% of patients a high level of intentional non-adherence.

Our findings are in line with previous research showing the influence of beliefs about medicines on intentional non-adherence to treatment of various chronic diseases [9,30,37]. Patients with MS who had stronger beliefs that medicines in general are overused by doctors (β = 4.204, *p* = 0.011) more often displayed intentional non-adherence, which is in line with the results of studies conducted among patients with HIV [37]. In contrast, in patients with hypertension [30], higher scores for necessity were associated with more non-adherence to treatment (r = 0.174, *p* = 0.003), while higher scores for overuse, harm and concerns were associated with less intentional non-adherence (respectively: r = −0.253, *p* < 0.001 vs. r = −0.336, *p* < 0.001 vs. r = −0.351, *p* < 0.001).

These findings demonstrate the diversity and uniqueness of different motivational factors which result in adherence behaviour that is not restricted to just drug-related factors like adverse effects. Instead, to more effectively target the improvement of adherence-related behaviour in people with MS, other psychological factors relating to the beliefs surrounding an illness or factors about the selection of treatment, alongside structural elements within healthcare systems, e.g., implementation support, and the societal environment, must be considered.

In many cases, it does not make sense for patients to use their prescribed medication if their condition is in an asymptomatic state and they generally feel well [37]. Therefore, it is important to conduct information campaigns indicating that only regular medication intake will reduce the risk of relapse. An assessment of the patient’s beliefs about medication should be routinely carried out [33], as this will identify patients who present, e.g., with medication-related concerns. By evaluating a patient’s beliefs concerning medication necessity and any concerns they may have, the quality of prescriptions made by clinicians may improve and help aid patients at being more engaged in their treatment decisions [8]. Provision of informational and emotional support by members of the treatment team can reduce concern and thus improve adherence.

However, for these measures to be carried out, there must be a positive relationship between doctor and patient. The basis for co-responsibility for the treatment process is communication between the doctor and patient. A well-informed patient will consciously make decisions regarding his or her own health and will better understand recommendations and the treatment process. Establishing rules of conduct with the treating physician leads to trust-building, to the therapeutic measures taken and improves adherence and thus increases the chances of achieving the expected health outcome.

## 5. Study Limitations

Although our study was carefully designed, a few limitations should be mentioned. Non-adherence to treatment was assessed based on the subjective opinion of the patients by using the Polish version of a specific questionnaire, which before was used only among elderly patients with hypertension. Another limitation was that the study was conducted in only one neurological centre in Wroclaw, so the results might be influenced by the quality of care in the university hospital, where the patients with MS are provided with coordinated care. Finally, this study is constrained because of its cross-sectional nature, which narrows definitive conclusions on whether medication-related beliefs have an effect on adherence among patients with MS. The longitudinal observation would provide more detailed information, which could be used in clinical practice. Future research conducted in multi-neurological centres is warranted to confirm or refute these findings.

## 6. Conclusions

To the best of our knowledge, this is the first Polish study to look at levels of intentional non-adherence in MS patients. A patient’s beliefs about the overuse of drugs and the harm they may bring have the most influence on non-adherence. Medical staff working with MS patients need to be aware of the possibility of non-adherence. A greater number of support and coping strategies must be developed for individuals in order to increase adherence to therapy in MS patients. The patient needs clear information about the treatment, discussing the value of drugs and the appropriateness of taking it despite the absence or low severity of disease symptoms. Education about the management of multiple sclerosis drugs among the patients is the best option to improving adherence. The supporting program should be based on an internet or monitoring system (SMS or alarm reminders). Furthermore, family plays an important role in the adherence process. In the future, artificial intelligence (AI) interventions can increase the effectiveness of medication adherence intervention programs.

## Figures and Tables

**Table 1:** Characteristics of the study group.

Variables		(n = 148)
Age [years]	mean ± SD	43.9 ± 10.19
	median	42
	quartiles	37–50
EDSS [score]	mean ± SD	2.38 ± 1.27
	median	2
	quartiles	1.5–3
Duration of the disease [years]	mean ± SD	12.07 ± 6.35
	median	11.5
	quartiles	7–17
Number of relapses in the last two years?	mean ± SD	0.62 ± 1.08
	median	0
	quartiles	0–1
Duration of DMTs use (years)	mean ± SD	6.22 ± 3.67
	median	6
	quartiles	0.3–9
Gender, n (%)	Female	100 (67.57%)
	Male	48 (32.43%)
Form of the medicine, n (%)	Injections	61 (41.22%)
	Tablets	87 (58.78%)
Place of residence, n (%)	Village	35 (23.65%)
	City < 50 thousand residents	40 (27.03%)
	City 50–500 thousand residents	20 (13.51%)
	City > 500 thousand residents	53 (35.81%)
Education, n (%)	Basic or vocational education	23 (15.54%)
	Secondary education	55 (37.16%)
	Higher education	70 (47.30%)
Marital status, n (%)	Single	30 (20.27%)
	Married	101 (68.24%)
	Divorced	16 (10.81%)
	Widowed	1 (0.68%)
Occupational activity, n (%)	Active/working	101 (68.24%)
	Retired	12 (8.11%)
	Pension	23 (15.54%)
	Unemployed	10 (6.76%)
	Student	2 (1.35%)
Complains, n (%)	Speech disorders	19 (12.84%)
	Hypertonia	33 (22.30%)
	Mood disorders	44 (29.73%)
	Sensory disorders	32 (21.62%)
	Mobility and balance disorders	84 (56.76%)
	Sexual disorders	39 (26.35%)
	Vision disorders	73 (49.32%)
	Sphincter disorders	52 (35.14%)
	Dysphagia	12 (8.11%)
	Fatigability	105 (0.95%)

SD—standard deviation, EDSS—Expanded Disability Status Scale, DMTs—disease modifying therapies, n—number of patients.

**Table 2:** INAS and BMQ questionnaire results.

		Mean	SD	Median	Min	Max	Q1	Q3
INAS		51.41	27.83	47.0	22	110	22.00	66
BMQ	Overuse	11.28	2.50	11.5	5	20	9.25	13
BMQ	Harm	10.69	2.55	11.0	4	20	9.00	12
BMQ	Necessity	18.30	3.41	18.0	7	25	16.00	21
BMQ	Concerns	14.17	3.29	14.0	5	21	12.00	16

SD—standard deviation, INAS—the Intentional Non-Adherence Scale, BMQ—the Beliefs about Medicines Questionnaire, Q1—the first quartile, Q3—the third quartile.

**Table 3:** Results of the univariate linear regression analysis.

Variable		Parametr	95% CI		*p*
Gender	Female	ref.			
	Male	7.406	−2.129	16.941	0.13
Age [years]		0.348	−0.091	0.788	0.123
Form of the medicine	Injections	ref.			
	Tablets	7.648	−1.408	16.703	0.1
EDSS score		0.142	−3.406	3.69	0.938
Place of residence	Village	ref.			
	City < 50 thousand residents	6.718	−5.94	19.375	0.3
	City 50–500 thousand residents	−1.207	−16.536	14.122	0.878
	City > 500 thousand residents	−1.387	−13.299	10.524	0.82
Education	Basic or vocational education	ref.			
	Secondary education	−0.236	−13.741	13.27	0.973
	Higher education	−7.859	−20.931	5.213	0.241
Marital status	Single	ref.			
	Married	−2.477	−13.778	8.824	0.668
	Divorced, Widowed	10.184	−6.315	26.684	0.228
Occupational activity	Active/working, student	ref.			
	Retired	16.141	−0.374	32.657	0.057
	Pension	9.388	−3.099	21.875	0.143
	Unemployed	1.775	−16.159	19.709	0.846
Disease duration (years)		0.098	−0.612	0.808	0.787
Number of relapses in the last two years?		1.101	−3.083	5.285	0.607
Duration of DMTs use (years)		0.339	−0.978	1.657	0.614
Speech disorders	No	ref.			
	Yes	4.055	−9.378	17.488	0.555
Hypertonia	No	ref.			
	Yes	0.215	−10.593	11.024	0.969
Mood disorders	No	ref.			
	Yes	−4.82	−14.631	4.992	0.337
Sensory disorders	No	ref.			
	Yes	−6.342	−17.222	4.538	0.255
Mobility and balance disorders	No	ref.			
	Yes	−1.446	−10.524	7.632	0.755
Sexual disorders	No	ref.			
	Yes	−3.515	−13.711	6.681	0.5
Vision disorders	No	ref.			
	Yes	−8.277	−17.175	0.621	0.07
Sphincter disorders	No	ref.			
	Yes	−1.551	−10.972	7.869	0.747
Dysphagia	No	ref.			
	Yes	2.634	−13.842	19.11	0.754
Fatigability	No	ref.			
	Yes	−3.907	−13.796	5.981	0.44

*p*—univariate linear regression.

**Table 4:** Results of the multivariate linear regression analysis.

Variable		Parametr	95%CI		*p*
Gender	Female	ref.			
	Male	8.599	−0.68	17.877	0.072
Age	[years]	0.137	−0.347	0.622	0.58
Form of the medicine	Injections	ref.			
	Tablets	7.896	−1.041	16.834	0.086
Occupational activity	Active/working, student	ref.			
	Retired	11.632	−6.272	29.537	0.205
	Pension	7.226	−5.24	19.693	0.258
	Unemployed	5.642	−11.469	22.753	0.519
Vision disorders	No	ref.			
	Yes	−6.497	−15.22	2.226	0.147
BMQ: Overuse		4.204	0.997	7.411	0.011 *
BMQ: Harm		−0.001	−3.223	3.222	1
BMQ: Necessity		0.244	−1.05	1.538	0.712
BMQ: Concerns		−1.365	−2.866	0.136	0.077

*p*—multivariate linear regression; * statistically significant (*p* < 0.05).

## Data Availability

Data are contained within the article.
